# Citizen-Patient Involvement in the Development of mHealth Technology: Protocol for a Systematic Scoping Review

**DOI:** 10.2196/16781

**Published:** 2020-08-28

**Authors:** Jorunn Bjerkan, Bridget Kane, Lisbeth Uhrenfeldt, Marit Veie, Mariann Fossum

**Affiliations:** 1 Faculty of Nursing and Health Science Nord University Levanger Norway; 2 Karlstad University Karlstad Sweden; 3 Faculty of Nursing and Health Science Nord University Bodø Norway; 4 Danish Center of Systematic Review: a Joanna Briggs Institute Centre of Excellence, Centre of Clinical Guidelines Aalborg University Aalborg Denmark; 5 Centre for Caring Research – Southern Norway Department of Health and Nursing Sciences Faculty of Health and Sport Sciences, University of Agder Grimstad Norway

**Keywords:** eHealth, mHealth, medical informatics, telemedicine, system development, patient participation, community participation, delivery of health care, person-centered, community-based participatory research

## Abstract

**Background:**

The development of mobile technology for information retrieval and communication, both at individual and health organizational levels, has been extensive over the last decade. Mobile health (mHealth) technology is rapidly adapting to the health care service contexts to improve treatment, care, and effectiveness in health care services.

**Objective:**

The overall aim of this scoping review is to explore the role of citizen-patient involvement in the development of mHealth technology in order to inform future interventions. By identifying key characteristics of citizen-patient involvement in system development, we aim to improve digital communication and collaboration between health care providers and citizen-patients, including sharing of health care data.

**Methods:**

The systematic scoping review will follow the Joanna Briggs Institute methodology for scoping reviews by searching literature in 3 steps. We will include literature reporting on the public, citizens, and patients participating in the development of mobile technology for health care purposes in MEDLINE, CINAHL, Scopus, EMBASE, and ProQuest Dissertations and Theses. A preliminary search was completed in MEDLINE and Scopus. The screening process will be conducted by 2 of the authors. Data will be extracted using a data extraction tool prepared for the study.

**Results:**

The study is expected to identify research gaps that will inform and motivate the development of mHealth technology. The final report is planned for submission to an indexed journal in November 2020.

**Conclusions:**

To our knowledge, this review will be the first review to provide knowledge about how citizen-patients participate in system developments for mHealth tools and the value that such involvement adds to the system development process.

**International Registered Report Identifier (IRRID):**

PRR1-10.2196/16781

## Introduction

### Background

The development of mobile technology for information retrieval and communication, both at individual and organizational levels, has been extensive over the last decade. Electronic or digital technology is rapidly adapting to both mobile platforms and health care service contexts to improve treatment, care, and effectiveness in health care services. These technologies are sometimes referred to as telemedicine, health information systems, and, more recently, eHealth [1,2]. Mobile health (mHealth) technology has emerged from eHealth as mobile technological platforms expand for wireless web-based communication and are designed and developed in an appropriate mHealth interface context [3,4]. As mHealth is still quite new, there are few definitions of the concept [5]; all of the definitions describe the relationship or extension of mHealth from eHealth. The World Health Organization introduced this definition: “Mobile Health (mHealth) is an area of electronic health (eHealth) and it is the provision of health services and information via mobile technologies such as mobile phones and Personal Digital Assistants (PDAs)“ [6]*.* mHealth platforms include tablet PCs and mobile smartphones. Programs or apps are being developed for these platforms and transformed through a new interface from existing eHealth applications or developed as new technologies. The use of such mobile apps makes access to health care information and communication unbound by time or place and potentially available for citizens and patients. The use of mHealth might be to improve or support health care or treatment for citizens, both within the public health domain and for marketing and sale in a commercial market [7,8]. Health care apps that are easily accessible to citizens and patients are generally welcomed and promoted by health authorities [9,10]. On the other hand, digital technology available for citizen-patients has not always been a success for end users. Both the lack of appropriate user interfaces and economic barriers have, until recently, also restricted the system adoption [11].

Political movements have, for several decades, influenced a demand for and the development of democratization and individualization in public policy in Western countries, which has also influenced health care service towards patient-empowered services [12,13]. mHealth development has further prompted new directions in health care policy. Health care models like person-centered health care emphasize a patient- and user-centered approach, regarding the patient or citizen as an equal or even dominant party to the care being offered [14,15]. Citizens are unlikely to assume a traditional passive patient role in which the health care provider (HCP) is the indisputable authority that is not open to argument [12,16]. The internet facilitates knowledge about health conditions and makes opportunities for communication potentially available to the public. There is now more opportunity for patients to influence health care [17]. Thus, the use of mHealth apps allows for the opportunity to support citizen-patient involvement and patient empowerment on health-related issues, even though many eHealth applications still lack a focus on patient involvement and empowerment [2,18]. The design and development process for new apps might take political influence into account by understanding the importance of including the public and patients in their development processes and mapping and meeting their needs, including system functionality and interface requirements [19,20]. On the other hand, in spite of public trends and politics as we describe, new technologies may still be launched with little or no citizen-patient involvement in the system development phases, hitting the user’s needs by chance or by parameters other than user participation strategies, as shown by Risling and colleagues [2]. Citizen-patient involvement may be emphasized for political as well as commercial reasons. However, if a tool developed is not used or if a system vendor does not hit the core target for requirements and needs of the public, commercial success will not be possible [21,22]. From a political viewpoint, a health care authority would benefit from citizen-patient involvement by reaching their target groups, if only for the purpose of improving health conditions in the population. Here, citizen-patient involvement may also be a matter of democracy and further empowerment, that is, being involved as a citizen [23,24].

There are many ways to involve system end users methodologically in the design and development process of digital technology. These methods can involve working with users directly in the requirements identification phase, through evaluation of prototypes, and through user evaluation and testing of the finished product [25]. The outcomes from both commercial and political strategies for citizen-patient involvement in system development and system use are expected to increase empowerment of the public as a whole. Any citizen involved in the use and development of the actual mHealth tools will likely experience empowerment. The successful use of mHealth relies on whether people want to, like to, and are able to use the apps offered [26-28].

A preliminary search in *JBI Database of Systematic Reviews and Implementation Reports*, Cochrane, MEDLINE, and Scopus returned a handful of reviews and review protocols covering the scope of mHealth [7,29-33]. The majority of the studies focused on technological issues or mHealth used for or by specific patient groups or medically diagnosed groups. An example of a technological approach is seen in the paper by Silva et al [7], which presents a methodological review that summarizes the state of the art of mHealth solutions. Silva et al [7] show the top mHealth apps available in the market in 2015 and discuss future strategies in mHealth development. Iribarren and colleagues [30] focus on text-messaging intervention platforms for mHealth applications. They also highlight the knowledge necessary for the development of technology that integrates text messaging in health intervention and research. Further, they discuss the advantages and disadvantages of the included platforms. Shah and Chiew [32] analyze design and usability of mobile apps for pain management but not if and how users were involved in the development of these mobile tools. Vo et al [33] focus on the strengths and weaknesses of the use of mHealth apps, showing increased health care engagement and empowerment for patients actively using such apps. Weaknesses found related to trustworthiness, appropriateness, personalization, and accessibility of the tools. None of the studies identified have citizen-patient involvement in system development as a specific focus area. Our study will differ from existing reviews, as our scope is not limited to patients in illness categories, nor does it limit the use of mHealth to any specified reasons. Specifically, our review will address the role of citizen-patients and their involvement in the development of mHealth.

### Aim and Review Questions

The overall aim of the scoping review is to explore the role of citizen-patients in the development of mHealth in order to inform future eHealth interventions. By identifying key characteristics of citizen-patient involvement in system development, we aim to improve digital communication and collaboration between HCPs and citizen-patients, including sharing of health care data.

Our review questions include the following: (1) How is the concept of citizen-patient involvement defined in the literature? (2) What research methods are used in the involvement of citizen-patients in the development of mHealth? (3) What are the advantages, disadvantages, and added value of citizen-patient involvement in the development of mHealth? (4) What are the challenges of involving citizen-patients in the different stages of the mHealth development process? and (5) What types of mHealth are identified in the literature?

### Inclusion and Exclusion Criteria

#### Participants

The review will consider papers that include the public or citizens in general and patients or users in particular having access to mHealth technology, including (1) citizens and patients across their lifespan, (2) citizens as patients, (3) citizens and patients using mHealth, (4) citizens as next of kin in their participation in the development or use of mHealth, and (5) citizens and patients participating in the development of mHealth.

For some patient groups, next of kin, spouses, significant others, children, or parents will be users of such technology because of the context or age of the patient. Examples include young children and older or cognitively impaired adults in need of assistance who make use of mHealth. Only studies that include citizens acting as private persons will be included in our study. In other words, HCPs or stakeholders participating in the development of mHealth technology will be excluded.

#### Concept

The concept of the papers included in this review is the involvement of the public, citizens, and patients in system design or system development of mHealth technology.

#### Context

The context of the papers included in this review is any demographic or geographic setting, in accordance with the worldwide use of mobile phones and other mobile platforms.

The same mHealth apps may be available in different health care situations, providing information on a general basis everywhere. The study will consider the inclusion of any setting where citizen-patients contribute to system development of mobile apps for health information both as receivers and senders. We aim to describe how, when, or in which stages of the development processes participation takes place.

#### Types of Sources

We plan to include scientific peer-reviewed studies, dissertations, and conference proceedings. All scientific research approaches and types of scientific study design will potentially be included in this review. The study might also include opinion papers from scientific journals but will not include magazines or newspapers due to the anticipated large number of papers and low level of scientific validity. mHealth is quite new and is a rapidly evolving technology. Because of this, the review will not restrict its search for papers by year of publication. Studies in Scandinavian languages, English, and German will be examined for inclusion.

## Methods

In an effort to assist in standardizing the conduct and reporting of scoping reviews, as proposed by Tricco et al [34] and supported by the Joanna Briggs Institute, the systematic scoping review will follow the template of the Joanna Briggs Institute methodology for scoping reviews [35,36].

### Search Strategy

Following the Joanna Briggs Institute methodology for systematic scoping reviews [35], we will review literature according to the inclusion criteria in 3 steps.

First, we will conduct an initial search to explore terms and keywords for the searches.

Second, the identified terms and keywords will be applied to searches across the chosen databases, and the results will be systemized.

Third, the reference lists from all the identified papers will be examined in order to reveal additional material not found in the second step of the search. Literature will be included or excluded according to the aim and the inclusion criteria of our review.

### Information Sources

The information sources in this systematic scoping review will include MEDLINE, Cumulative Index to Nursing and Allied Health Literature (CINAHL), Scopus, EMBASE, and ProQuest Dissertations and Theses. Keywords for the initial search and the strategy for searching is shown in an example from two of the selected databases, MEDLINE and Scopus, in Table 1. The first four example searches in Table 1 show the initial search strategy, and the fifth search is the combination of searches.

**Table 1:** Preliminary search on Scopus and MEDLINE.^a^

Search number	Query	Results on MEDLINE	Results on Scopus
1	Patient(s)^b^ OR user(s) OR citizen(s) OR citizen patient(s)	6,795,125	9,986,893
2	Patient(s) participation^b^ OR Community participation^b^ OR Crowdsourcing^b^ OR participation OR involvement OR engagement OR patient and public involvement OR PPI OR patient(s) involvement/engagement OR citizen(s) involvement/engagement/participation OR citizen patient(s) involvement/engagement/participation OR public involvement/engagement/participation OR user involvement/engagement/participation OR community involvement/engagement	683,863	1,245,902
3	Community-based participatory research^b^ OR Citizen science^b^ OR system development OR system design(s) OR system analyse/analysis OR participatory design OR participatory research OR development OR co-design OR community system(s)	2,134,092	6,914,266
4	Telemedicine^b^ OR Mobile application(s)^b^ OR Cell phone(s)^b^ OR Medical informatics^b^ OR Medical informatics application(s)^b^ OR Health information exchange^b^ OR Medical informatics computing^b^ OR mHealth OR eHealth OR electronic health OR mobile health OR health technology OR health informatics OR mobile phone(s) OR smartphone(s) OR mobile phone app(s) OR mobile health app(s) OR smartphone app(s) OR cell phone app(s)	95,279	266,291
5	#1 AND #2 AND #3 AND #4	1048	2433

^a^Ovid MEDLINE and Epub Ahead of Print, In-Process & Other Non-Indexed Citations, Daily and Versions 1946 to May 15, 2020.

^b^Medical subject heading term.

### Study Selection and Extraction

The Rayyan online software platform (Qatar Computing Research Institute) will be used to facilitate the entire screening process [37,38]. We will use the bibliographic system Endnote X9 (Clarivate Analytics) to collect and upload all identified citations. Duplicates will be removed. Titles and abstracts of the papers of current interest will then be assessed independently by 2 reviewers against the inclusion and exclusion criteria listed above. The assessment will be documented, guided by the Preferred Reporting Items for Systematic Reviews and Meta-Analyses template for flows of inclusion [39]. Potential papers for inclusion will undergo the same procedure, being assessed in detail against the inclusion criteria by 2 independent reviewers. Reasons for exclusion of studies will be recorded and reported in our scoping review publication. Data will be extracted from included papers by the use of a data extraction tool developed for the review study. Data to be extracted are shown in Textbox 1. The data extraction tool may be modified and revised during the data extraction process and eventual changes will be described in the final report. The extracted data will include relevant details about the population, concept, context, study methods, and key findings.

**Data to be extracted.**

**Study design**

- Authors
- Year
- Country
- Aim of the study
- Population/participants involved
- Data collection methods
- System development methods

**Study context and concept**

- Context of system development
- Intervention/type of system development
- Scale/size and duration of development project
- Stage of participation in system development
- Identified outcomes: challenges, values, advantages, and disadvantages

Any disagreements between the reviewers during the screening process will be resolved through discussion or by involving additional reviewers. The reviewers may also contact authors of papers to request missing or additional data. 

## Results

The extracted data will be presented to align with the objective of this systematic scoping review by using diagrammatic or tabular forms. The report will also include a narrative summary to accompany the tabulated or charted results in order to relate to the review’s objective. These collated results will finally be presented in a systematic scoping review publication. This systematic scoping review protocol was first initiated by Nord University in October 2018. The study is undertaken without any external funding. We commenced preliminary data collection in January 2019, which was updated in April 2020. We expect the final results to be submitted in a systematic scoping review in November 2020.

## Discussion

### Contribution to mHealth Development

The results from this scoping review will aim to inform a variety of stakeholders, including authorities and vendors, about involving citizen-patients in the development of mHealth. Such knowledge may improve the development process and results for future mHealth. The review may also inform authorities about the possible success of their mHealth strategies and the effects of involving citizen-patients in the development processes on the achievement of their goal [6]. The results from the review might also address any challenges explored in involving citizen-patients in mHealth development processes. Identified knowledge from this review would be valuable for future projects to improve any citizen-patient involvement in the development of mHealth technology processes instead of such involvement being minimized or downgraded. When researchers consider applying strategies like participatory design or participatory action research in their projects by actively involving citizen-patients, knowledge from the review may inform their research protocols and add value to their results.

### Conclusion

To our knowledge, this review will be the first systematic scoping review to provide knowledge about how citizen-patients participate in system developments for mHealth technology and the value that such involvement adds to the system development process. We aim to find answers to the review questions from the results of our analysis. This review will also seek to identify further research gaps and possible needs for further systematic reviews.

## Abbreviations

CINAHL: Cumulative Index to Nursing and Allied Health Literature
HCP: health care provider
mHealth: mobile health
